# Altered Achilles tendon morphology in individuals with chronic post-stroke hemiparesis: a case report

**DOI:** 10.1186/s12880-020-00431-0

**Published:** 2020-04-03

**Authors:** Jing Nong Liang, Kai-Yu Ho

**Affiliations:** Department of Physical Therapy, University of Nevada, Las Vegas, 4505 South Maryland Parkway, Box 453029, Las Vegas, Nevada 89154 USA

**Keywords:** Post-stroke hemiparesis, Walking speed, Sonography, Achilles tendon, Case report

## Abstract

**Background:**

Individuals post-stroke walk slowly and with more effort, which puts them at higher risks for falls. The slow walking speed results from insufficient propulsive forces generated by the paretic leg. Current rehabilitative efforts to improve walking function target increasing propulsive forces, but overlook the muscle-tendon unit.

**Case presentations:**

Two individuals with chronic post-stroke hemiparesis are presented. In both individuals post-stroke, paretic ankle plantarflexors presented with increased muscle tone. Gait kinetics revealed asymmetric propulsive forces, specifically, insufficient propulsive forces by the paretic legs, consistent with previous literature. Sonography revealed increased thickness of paretic Achilles tendon at the calcaneal insertion, in both stroke cases, in contrast to comparable Achilles tendon thickness between limbs in the non-neurologically impaired controls.

**Conclusion:**

Tendon unit integrity should be considered in individuals post-stroke who demonstrate abnormal muscle tone and insufficient propulsion during gait.

## Background

Stroke is a leading cause of adult long-term disabilities worldwide. While majority of individuals with post-stroke hemiparesis are able to regain walking function, there often remains the hallmark impairment of slow walking speed. Slower walking speed has been reported to increase fall risks in people post-stroke [1]. Insufficient propulsive forces has been identified as the major cause underlying slower walking speed [2]. As a critical component for successful execution of appropriate propulsive forces, the muscle-tendon unit has yet to be examined.

Abnormal forces applied to tendon tissues have been found to cause morphological changes similar to those observed in degenerative tendon. These changes include collagen fiber disorganization, increased water content, increased glycosaminoglycan content, thinner collagen fibers, reduced overall collagen content, increased type II collagen, and reduced tendon stiffness [3]. Using sonographic imaging, a number of previous studies have reported morphological changes (i.e. increased tendon thickness [4] and cross-sectional area [5]) in diseased tendons. Thus, although there is reason to believe the aforementioned abnormalities of the Achilles tendon can develop in individuals post-stroke due persistent increases in muscle tone, no data has been published to support this. In this case report, we examined muscle tone of the ankle plantarflexors, propulsive forces during walking, and thickness of the Achilles tendon in the paretic and non-paretic legs of individuals with chronic post-stroke hemiparesis compared with non-neurologically impaired controls.

## Case presentation

Two individuals with chronic post-stroke hemiparesis are presented. The first case (Stroke 1) is a 50-year old male who survived a left hemorrhagic cortical cerebral vascular accident 5.2 years ago. The second case (Stroke 2) is a 62-year old male who survived a right ischemic cortical cerebral vascular accident 5.6 years ago. Both individuals with chronic post-stroke hemiparesis have since regained independent community ambulatory function without the use of assistive devices, but reported slow walking speed despite considerable effort to propel forward. Two non-neurologically impaired individuals are presented as controls (Table 1).

**Table 1:** Participant characteristics

**Cases**	**Age (years)**	**Sex**	**Time Post Stroke (years)**	**Self-selected walking speed (m/s)**
**Post-stroke:**				
**Stroke 1**	**50.40**	**M**	**5.18**	**1.19**
**Stroke 2**	**62.06**	**M**	**5.64**	**1.29**
**Non-neurologically impaired control:**				
**Control 1**	**47.71**	**M**		**2.77**
**Control 2**	**45.99**	**F**		**2.33**

### Clinical and gait assessments 

Upon arrival of each participant, we assessed the muscle tone at the ankle joints, using the Modified Ashworth Scale. Each participant walked on an instrumented dual-belt treadmill at self-selected walking speed. Ground reaction forces were recorded.

### Imaging acquisition

**Fig. 1:**
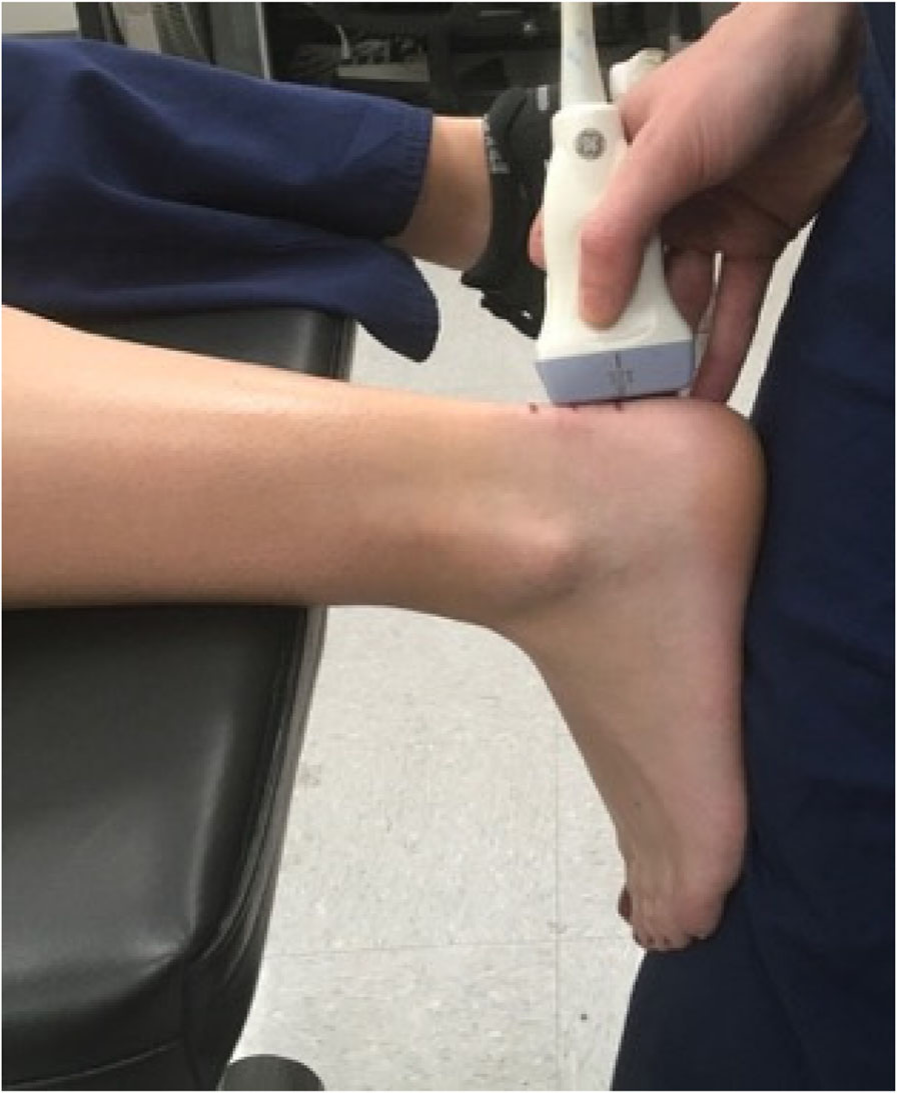
Placement of the ultrasound transducer

To examine tendon morphology, brightness-mode sonographic images were captured on bilateral Achilles tendons using a portable ultrasound imaging scanner (GE LOGIQ-e, GE Healthcare, Milwaukee, WI, USA). Achilles tendons were scanned while the individual lay prone with the knee in an extended position, and the ankle was passively maintained in a neutral position (Fig. 1). A linear array transducer (GE 12 L-RS, bandwidth 5-13 MHz, width 38.4 mm, GE Healthcare, Milwaukee, WI, USA) was used with the musculoskeletal preset at a depth of 2 cm. Images were captured in three locations longitudinally, centered at 0 (distal), 2 (mid-), and 4 (proximal) cm from the superior aspect of the calcaneus.

### Imaging processing

The tendon thickness was measured on each longitudinal image (i.e., proximal, mid-, and distal portions), which was defined as the perpendicular distance between the borders that outline the Achilles tendon. The distal thickness was measured at the Achilles tendon-calcaneaus intersection. For the proximal and mid-potions of the Achilles tendon, the thickness was measured at the center from the proximal and mid-portion images.

### Imaging reliability

To assess intra-rater reliability of tendon morphological measures, the sonographer performed repeated data collection on 5 participants, separated by 1 week. Intraclass correlation coefficients (ICCs) and standard errors of measurement (SEMs) were calculated. The investigator demonstrated excellent measurement reliability and low SEMs for tendon thickness (ICC = 0.984; SEM = 0.037 mm).

### Outcomes

In Stroke 1, the paretic gastrocnemius and paretic soleus muscles presented scores of 2 and 1 respectively on the Modified Ashworth Scale, indicative of increased muscle tone of the paretic limb. Gait kinetics revealed asymmetric ground reaction force profiles between paretic and non-paretic legs during walking, with 32.3% less peak propulsive forces generated by the paretic leg compared to that by the non-paretic leg. Similarly, in Stroke 2, the paretic gastrocnemius and paretic soleus muscles presented scores of 1 on the Modified Ashworth Scale. Gait kinetics revealed 64.3% less peak propulsive forces generated by the paretic leg compared to that by the non-paretic leg. This asymmetry in peak propulsive forces during walking is characteristic in individuals with chronic post-stroke hemiparesis, and has been identified as the major contributor to slower walking speed [2].

**Fig. 2:**
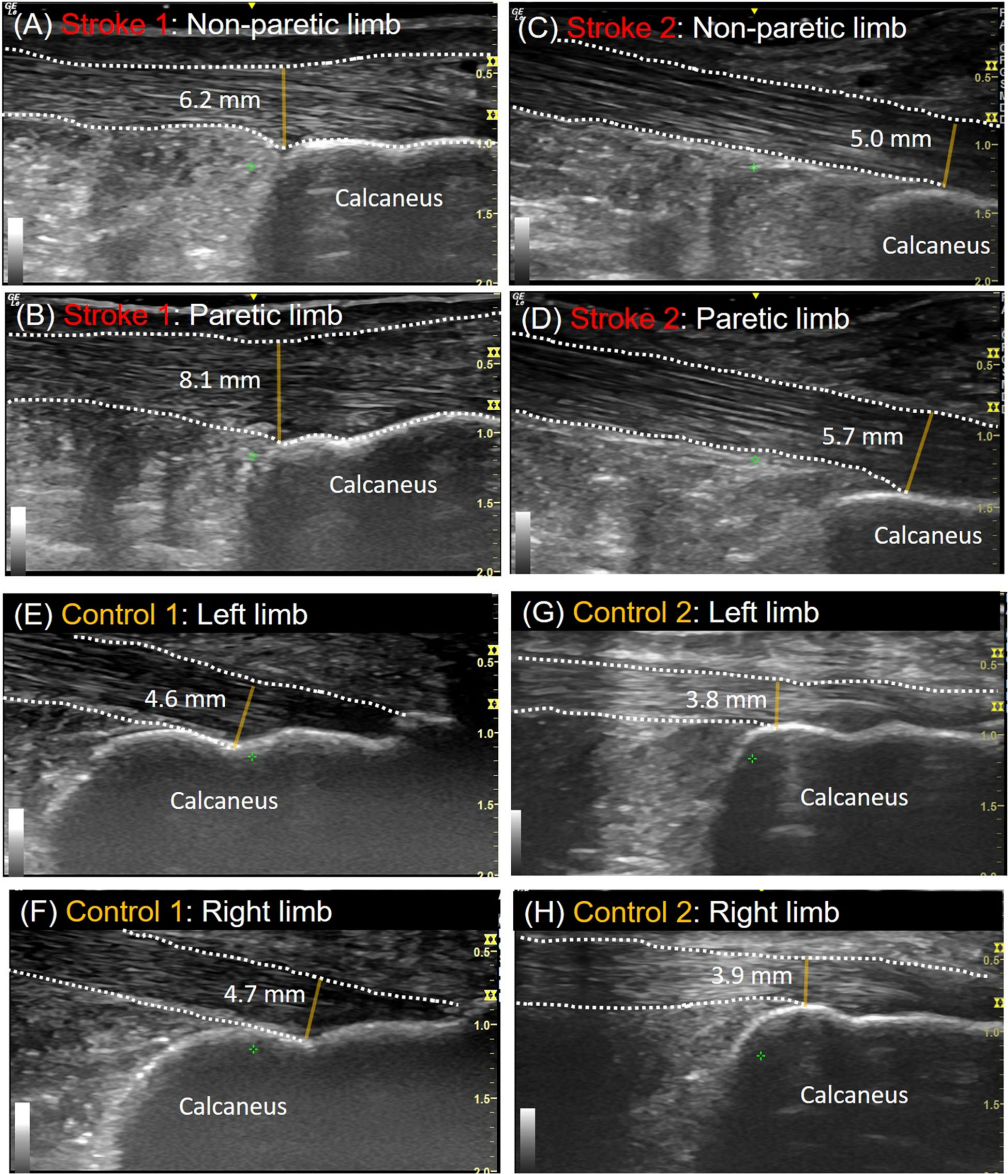
Longitudinal views of the Achilles tendon sonography at the Achilles tendon insertion for **a** the non-paretic limb of stroke case 1; **b** the paretic limb of stroke case 1; **c** the non-paretic limb of stroke case 2; **d** the paretic limb of stroke case 2; **e** the left limb of control case 1; **f** the right limb of control case 1; **g** the left limb of control case 2; **h** the right limb of control case 2. The yellow line on each image indicates the thickness of the Achilles tendon. The dash lines highlight the borders of the Achilles tendon

The sonographic data showed that the paretic limb revealed increased thickness of distal Achilles tendon (mean ± SD = 6.9 ± 1.7 mm) when compared to non-paretic limb (5.6 ± 0.8 mm) (Fig. 2a-d). We observed similar thickness in both paretic and non-paretic limbs for proximal (paretic limb: 5.4 ± 0.2 mm V.S. non-paretic limb: 5.7 ± 0.1 mm) and mid- (paretic limb: 5.0 ± 0.5 mm V.S. non-paretic limb: thickness: 5.0 ± 0.3 mm) portions of the Achilles tendon. For non-neurologically impaired controls, both sides exhibited comparable thickness for proximal, mid-, and distal portions of the Achilles tendon (Fig. 2e-h).

## Discussion and conclusions

In this report, we sought to explore the association between muscle tone of the ankle plantarflexors, propulsive forces during walking, and gross morphology of the Achilles tendon in persons with chronic post-stroke hemiparesis. As none of the individuals with post-stroke hemiparesis reported in this study reported any past or current symptoms/injuries of the Achilles tendon, the increased insertional thickness of the Achilles tendon is likely associated with chronic increases of the muscle tone of ankle plantarflexors in the paretic limb. Our observations suggest that altered morphology of the Achilles tendon in the paretic limb might contribute to the insufficient propulsive forces during gait, and thus the slow walking speed in an individual chronically post-stroke.

One factor that may have been overlooked in patients with post-stroke hemiparesis is altered muscle-tendon unit output force as a result of altered mechanical properties of the Achilles tendon. Post-stroke, due to altered descending input, increased muscle tone, particularly in the paretic ankle plantarflexors, is common, and negatively impacts locomotor control. In the cases presented, it is speculated that chronic muscle tone maladaptations in paretic ankle plantarflexors resulted in altered tendon morphology and composition, thereby leading to altered mechanical properties. Specifically, it has been found that focal tendon thickening and collagen fiber disorganization are related to reduced stiffness and elastic modulus of the tendon [6]. The more compliant tendon as a result of collagen fiber disorganization might compromise force transmission during gait [7], thereby leading to insufficient propulsive forces generated during gait, observed in these cases.

One limitation of this study is that the stroke participants presented in this case series were all chronically post-stroke, where morphological adaptations have already occurred. It would be important to explore the time course of adaptations. Additionally, future studies should address multi-level adaptations by examining the contribution of the altered neural circuitry post-stroke [8, 9].

Our findings can significantly impact physical therapy interventions for people post-stroke. Current rehabilitative efforts to improve walking speed should consider examining tendon integrity in individuals post-stroke who demonstrate excessive muscle tone and insufficient propulsive forces during gait. Since tendon adaptations contribute to altered walking mechanics, a treatment strategy aiming at improving the neuromuscular control should be employed. For instance, a treatment plan focusing on improving tendon health (e.g., eccentric exercises) [10] may be included in their rehabilitation program to improve walking.

## Data Availability

All data generated or analyzed during this study are included in this published article.

## Abbreviations

ICC: Intraclass correlation coefficients
SEM: Standard errors of measurement
